# Effect of caregiver characteristics on dementia management strategies

**DOI:** 10.1590/S1980-5764-2016DN1002010

**Published:** 2016

**Authors:** Débora Dalpai, Ramon Castro Reis, Analuiza Camozzato de Pádua

**Affiliations:** 1 Medical student at Universidade Federal de Ciências da Saúde de Porto Alegre (UFCSPA), Porto Alegre, Brazil, with FAPERGS scholarship scientific initiation.; 2 MD. Psychiatrist, MSc, Porto Alegre, Brazil.; 3 MD.PhD. Psychiatrist, PhD, professor of Psychiatry at Universidade Federal de Ciências da Saúde de Porto Alegre (UFCSPA), Porto Alegre, Brazil.

**Keywords:** dementia, management strategies, professional caregiver, burden

## Abstract

Caregiving has an important influence on the prognosis of dementia, particularly regarding the management strategy implemented. Therefore, evaluating which characteristics of caregivers can influence the choice of a particular strategy for managing dementia is needed.

**Objective:**

To evaluate the association between characteristics of caregivers and their management strategies as applied to patients with dementia.

**Methods:**

A cross-sectional study involving 45 professional caregivers from two nursing homes in Porto Alegre, Brazil, was conducted. Age, gender, education, years as a caregiver, income, burden, depressive and anxiety symptoms and dementia management strategies were evaluated for all participants. Pearson's or Spearman's correlation tests were applied according to the variable distribution (parametric or non-parametric). Bivariate correlation analysis was applied. P<0.05 was considered statistically significant.

**Results:**

There was a significant and moderate positive correlation between burden measured by the Zarit Burden Interview and criticism measured by the Dementia Management Strategies Scale (Spearman's rho = 0.555, p < 0.001). No other correlations were observed.

**Conclusion:**

Among the caregiver characteristics that directly affect the approach to managing dementia, high caregiver burden was found to be associated with high criticism, an authoritative way of managing dementia. This exploratory study indicated that a possible way of decreasing negative dementia management is to reduce caregiver burden.

## RESUMO

A forma de cuidar o paciente tem uma influência importante no prognóstico da demência, principalmente no que diz respeito às estratégias utilizadas por cuidadores para manejar sintomas comportamentais destes pacientes. Assim, faz-se necessário avaliar quais características dos cuidadores poderiam influenciar na escolha de uma estratégia específica para manejo da demência.

**Objetivo:**

Avaliar a correlação de variáveis dos cuidadores com as diferentes formas de manejo da demência.

**Métodos:**

Estudo transversal, com 45 cuidadores profissionais de duas instituições de longa permanência para idosos de Porto Alegre, Brasil. Idade, gênero, escolaridade, nível socioeconômico, anos de trabalho como cuidador, sobrecarga de cuidado, sintomas ansiosos e depressivos e estratégias de gerenciamento da demência foram avaliados. Foram utilizados os testes de correlação de Pearson ou Spearman, de acordo com a distribuição das variáveis (paramétricas ou não-paramétricas). Foi considerado como estatisticamente significativos valores de p < 0,05.

**Resultados:**

Houve correlação positiva moderada e significativa entre sobrecarga de cuidado pela Escala de Zarit e criticismo pela Escala de Estratégia de Gerenciamento da Demência (Spearman rho = 0,555, p < 0,001). Não foram observadas outras correlações.

**Conclusão:**

Entre as características dos cuidadores que afetam diretamente a forma de manejo da demência, percebemos que a sobrecarga de cuidado foi associada ao alto criticismo, uma forma autoritária de manejo da demência. Este estudo exploratório aponta que uma possível maneira de reduzir estratégias negativas de gerenciamento da demência é reduzir a sobrecarga do cuidador.

## INTRODUCTION 

Caregiving approaches and care management strategies have been associated with a decrease in neuropsychiatric symptoms of dementia by modifying the environment.^1^ Furthermore, caregiver coping, i.e., the strategies used to manage stress derived from the caring for dementia patients, may also influence other outcomes in dementia. In a longitudinal population-based study, the regular use of problem-focused coping by caregivers was associated with slower decline in patient cognition and function.^2^ In general, coping strategies are theorized as active or avoidance strategies. Examples of active strategies include reframing the problem, looking on the bright side, seeking support, finding solutions coupled with formulating and implementing a plan of action to address the problem, whereas ignoring the problems is an avoidance strategy.^3^

The coping strategy used when caring for persons with dementia was also associated with caregiving burden. Positive reappraisal of the situation, an active coping style, was negatively correlated with burden in a sample of one hundred and seventy-two caregivers of patients suffering from Alzheimer's type dementia.^4^ A systematic review showed that dysfunctional coping (strategies such as self-blame, denial and avoidance) correlated with higher levels of anxiety and depression in caregivers while emotional support and acceptance-based coping correlated with less anxiety.^5^ One review investigated factors that predict caregivers' coping strategies. It showed that younger and more highly educated caregivers, who had greater emotional support and knowledge of dementia used mostly solution-focused coping, while caregivers with less emotional support exhibited more dysfunctional coping.^6^

A scale for evaluating the specific management strategies of caregivers of dementia patients was developed by Hinrichsen & Niederehe (1994) who proposed three strategies: criticism, encouragement and active management.^7^ Criticism involves authoritative attitudes by the caregiver toward the elderly including yelling, threatening, criticizing and related behaviors. Encouragement includes efforts to praise the patient, stimulate discussion about patient feelings and demonstrate to him or her the best view of everything in life. Active management consists of safeguarding, monitoring and assisting the patient by modifying the environment and daily routine. 

Few studies have examined whether these three strategies (criticism, encouragement and active management) are correlated with higher burden of caregiving. In the original study, the use of active management and criticism was associated with greater caregiver burden.^7^ Similarly, the use of criticism as a coping strategy was one of the significant predictors of burden in one hundred and seven Asian family caregivers of individuals with dementia. In the same study, the use of encouragement as a specific dementia management strategy was the only significant predictor of the caregiver's subjectively perceived gains in relation to the caregiving experience.^8^

Given this scenario, more studies investigating whether these specific dementia management strategies are related to burden and to demographic or clinical characteristics of caregivers are required, and this was the aim of the current investigation. The evaluation of these correlations is relevant, since some of these caregiver characteristics may be amenable to interventions aimed at improving dementia management and consequently achieving better outcomes for patients.

## METHODS

A cross-sectional study involving a convenience sample of all professional caregivers from two of the largest nursing homes in Porto Alegre, a southern Brazilian city, was conducted. Participants were recruited according to the follow inclusion criteria: age 18 years or older, educational level ≥ 4 years of study, a minimum of 30 hours/week of direct care with dementia patients, and agreed to and signed the informed consent form for participation. Of the 76 professional caregivers that met the inclusion criteria, 31 (41%) refused to participate and therefore 45 were included in the study. Data were collected from September 1^st^ until December 2012, spanning 4 months. 

Age, gender, education, marital status, family income, years as caregiver, burden, depressive and anxiety symptoms, and dementia management strategies were evaluated for all participants. Two previously trained members of the research team (a medical student and a psychiatrist) applied the measurement instruments. A brief description of these scales is outlined below:

*Sociodemographic questionnaire:* this included gender, age, education (years of study) and the index of family purchasing power (income) of the Brazilian Association of Research Companies Socioeconomic Survey. This index evaluates the number of family consumer goods such as radios, televisions, DVDs, washing machines and cars; the number of bathrooms in the house; the number of housemaids in the family and the educational level of the head of the family. The total score corresponds to different categories of family income. Corresponding categories for continuous scores are: Class E (0-7), D (8-13), C2 (14-17), C1 (18-22), B2 (23-28), B1 (29-34), A2 (35-41), A1 (42-46). This level increases from low to high family income.^9^

*Zarit Burden Interview - ZBI*: this 22-item scale evaluates the impact of caring on psychological well-being and social life of the caregiver. Each item is rated from 0 to 4 and higher scores indicate higher burden. Total scores of 0-20 indicate none or minimum burden, 21-40 low to medium burden, 41-60 medium to severe burden and 61-88 severe burden.^10^ ^,^ ^11^

*Beck depression inventory (BDI):* this self-administered questionnaire evaluates depressive symptoms that have occurred during the month leading up to application of the scale. It has 21 items whose values range from 0 (I do not feel it) to 3 (I do feel it and I can't stand it anymore). A total score of 17-20 indicates borderline clinical depression, 21-30 moderate depression, 31-40 severe depression, and over 40 extreme depression.^12^

*Beck anxiety inventory (BAI)*: this is a self-administered questionnaire with 21 items representing the most common anxious symptoms observed in clinical practice. It measures symptoms that have occurred during the month leading up to application of the scale. Each item is rated from 0 (not at all) to 3 (severely). Total score is the sum of all rated items. A total score of 0 - 7 is interpreted as minimal level of anxiety; 8 - 15 as mild; 16 - 25 as moderate; and 26 - 63 as severe.^13^

*Dementia Management Strategy Scale - DMSS*: this 28-item scale measures the ways in which the caregiver manages dementia problems presented by patients. These strategies are categorized into criticism (11 items), encouragement (8 items) and active management (9 items). Each item of the three subscales is rated from 1 (never) to 5 (most of the time). The DMSS measures the frequency with which a caregiver employs specific strategies tied to behavioral problems evidenced by the dementia patient. Scores for the three DMSS subscales were derived by summing the raw score values for each of the included items rather than calculating factor scores. Each DMSS subscale is rated independently, measuring the frequency (never to most of the time) that the caregiver employs each different strategy. The maximum scores are 55, 40 and 45 for criticism, encouragement and active management, respectively. Yelling, criticizing, threatening and related behaviors are considered criticism items, while efforts to praise the patient, get him or her to discuss feelings or look on the bright side of things are encouragement strategies. Activities to safeguard, assist, engage, stimulate, monitor and associated behaviors primarily directed toward modifying the environment or daily routine are part of the active management category.^7^ ^,^ ^14^

Ethical aspects. The research project was approved by the Research Ethics Committee of the UFCSPA (number 11-830). All participants signed the informed consent form for study participation after receiving explanations about the project.

Statistical analyses: descriptive analyses for all variables were initially carried out. The correlation of each dementia management strategy (criticism, encouragement and active management) with age, education, income, years as a caregiver, burden, depressive and anxiety symptoms was evaluated by Pearson's or Spearman's correlation tests according to the variable distribution (parametric or non-parametric). Marital status of caregivers was analyzed by Student's *t-*test. SPSS v17 software was used as the statistical package. P<0.05 was considered statistically significant.

## RESULTS

Table 1 shows descriptive data for age, gender, education, marital status, income, years as caregiver, total score on DMSS subscales, ZBI, BAI and BDI scales. 

**Table 1:** Demographic, professional and clinical characteristics of caregivers.

Variable		Descriptors	Range
Age*		38.2/9.6	19-64
Gender**	Female	100	
Education (years of study)		13 (13;13)	3-15
Marital status**	Married	53	
	Not married	47	
Family income*		23.6/4.9	
Professional experience (years as caregiver)^#^		4 (1.6; 12.5)	0.25-32
DMSS - criticism*		25.1 (5.9)	13-40
DMSS - encouragement*		28.5 (4.2)	18-38
DMSS - active management*		33.7 (3.8)	26-42
Zarit Burden Interview ^#^		18 (13.5; 23)	6-40
Beck Anxiety Inventory^#^		6 (1.5; 10)	0-30
Beck Depression Inventory^#^		5 (1; 7)	0-21

*Expressed as median and SD; **Expressed as percentage; ^#^Expressed as median (p25;p75).

DMSS: Dementia Management Strategy Scale.

Most caregivers (97.8%) had low or medium family income, with 19 (42.2%) caregivers having low, 25 (55.6%) medium and 1 (2.2%) high family incomes. The scores for the three DMSS subscales were independent, with different total sums, precluding comparisons. Caregivers had none or minimum burden scores on the ZBI, minimum anxiety on the BAI, and no clinical depression on BDI scales. 

Table 2 shows the correlation between the three dementia management strategies and modifiable caregiver characteristics. DMSS criticism showed a significant and moderate positive correlation with ZBI score. No other correlations were observed. 

**Table 2:** Coefficient of correlation between dementia management strategies and caregiver characteristics.

	DMS criticism	DMS encouragement	DMS active management
Age*	-0.087	-0.014	-0.173
Education**	0.041	0.090	-0.072
Income*	-0.288	-0.099	-0.016
Professional experience**	0.170	0.077	0.058
Zarit Burden Interview**	0.555^+^	0.265	0.276
Beck Depression Inventory**	0.180	-0.046	-0.104
Beck Anxiety Inventory**	0.128	-0.260	-0.221

^+^p<0.05. *Pearson's correlation coefficient; **Spearman's rho correlation coefficient; DMS: Dementia management strategy.

There were no significant differences in criticism, encouragement or active management DMSS sub-scale scores between married and unmarried caregivers (p=0.073; p=0.291; p=0.284, respectively, by Student's *t-*test).

## DISCUSSION 

The objective of this study was to evaluate the correlation between modifiable demographic and clinical characteristics of caregivers and three different dementia management strategies: active management, encouragement and criticism. We found a significant and moderate positive correlation between burden and criticism. The higher the caregiver burden, the higher the use of criticism as a dementia management strategy. Our result partially agreed with previous studies associating the use of criticism with greater burden,^7^ ^,^ ^8^ although these involved family caregivers. Hinrichsen & Niederehe (1994) noted that family members who more frequently utilize critical, coercive or authoritarian approaches with the patient are also those who experience considerable frustration and emotional upset,^7^ where this is one of the possible hypotheses explaining our result. In this regard, there is a lack of data available for professional caregivers. 

There are few studies comparing informal with professional caregivers.^15^ A study of Takahashi et al. (2005) examined differences in depressive state and associated factors between informal and professional caregivers. The study revealed greater age, more extended duration of care, higher care burden, lower quality of life and more frequent depression in informal caregivers.^15^ In addition, another investigation showed that caregivers who reported poor family functioning had higher ratings of strain and burden.^16^ Thus, it can be hypothesized that these differences between informal and professional caregivers could influence the choice of dementia management strategy with family caregivers having more difficulties managing neuropsychiatric symptoms of dementia.

The cross-sectional design precluded further exploration of the potential causal relationship between burden and use of criticism as a dementia management strategy. Our hypothesis is that criticism is one of the common strategies to deal with burden. However, as criticism tends to worsen patient symptoms (which in turn may increase burden), a vicious cycle can be created. In other words, the behavior of each part of the caregiver-patient dyad directly influences response of the other, producing a reciprocal phenomenon or feedback loop of mistreatment.^17^ ^,^ ^18^

Besides the association with criticism, caregiver burden has previously been associated with avoidance coping^19^ and abuse.^20^ Caregiver burden is also a significant predictor for the death or institutionalization of patients with dementia.^21^ Therefore, these findings reinforce the importance of being attentive to burden, previously described as accessible to intervention.^22^ Some types of intervention were able to reduce caregiver burden, such as psychoeducational,^23^ ^,^ ^24^ psychosocial,^25^ person-centred^26^ and interventions focusing on coping strategies.^27^

The measurement of management strategies for patients with dementia using a specific scale was a possible limitation of the present study, since professional attitudes and behaviors not self-perceived were impossible to detect. Direct observation could have been a possible alternative. However, this approach also has limitations, as caregivers may appear competent in demonstrating a management strategy when observed, but not during usual care.

We could presume that caregiver demographic characteristics such as age, gender, marital status, education and family income impact their strategies for managing behavioural symptoms of patients with dementia. However, no correlation was found between any of these variables with some of the dementia management strategies evaluated, although these results should be interpreted with caution. Our sample included 45 female professional caregivers and therefore the association with sex was not evaluated. In addition, most caregivers had low or medium family incomes, where the low variability of these scores may also have impacted our results. Further studies on this issue with larger samples including caregivers with higher income should be conducted. Age and education of caregivers were not associated with any of the dementia management strategies. A possible explanation for these findings is that the dementia management strategies are more impacted by formal training programs addressing this issue than by general education. Another hypothesis is that a specific type of dementia management strategy may be more linked to psychological aspects of the caregiver, such as burden, as found in the present study. Furthermore, these demographic variables might be associated with dementia management strategies that were not measured in this study, as commented above. 

This was an exploratory study with a small sample. Notwithstanding these limitations, the significance of this study involves the investigation of an important aspect that influences the treatment and quality of life of patients with dementia living in nursing homes: caregiving. 

 Being attentive to caregivers and taking care of them can strengthen the relationship between caregiver and patient, benefitting both parties. This study evaluated professional caregivers working in a nursing home. The association of caregiver burden with greater use of criticism as a dementia management strategy by these professionals highlights the importance of focusing on the institutional work environment and mental health of professionals. Furthermore, improving caregiving seems to be a possible form of improving the natural course of dementia, the working environment, the health of caregivers and possibly the costs of professional caregiving.
